# A case of enoxaparin-induced thrombocytopaenia during treatment of acute myocardial infarction

**DOI:** 10.5830/CVJA-2016-010

**Published:** 2016

**Authors:** Snag Yup Lim, Se Ryeon Lee, Yong Hyun Kim, Jin Seok Kim, Seong Hwan Kim, Jeong Chun Ahn, Woo Hyuk Song

**Affiliations:** Department of Internal Medicine, Ansan Hospital, Korea University, Dan Won-Gu, Ansan, GyoungGi-Do, Korea; Department of Internal Medicine, Ansan Hospital, Korea University, Dan Won-Gu, Ansan, GyoungGi-Do, Korea; Department of Internal Medicine, Ansan Hospital, Korea University, Dan Won-Gu, Ansan, GyoungGi-Do, Korea; Department of Internal Medicine, Ansan Hospital, Korea University, Dan Won-Gu, Ansan, GyoungGi-Do, Korea; Department of Internal Medicine, Ansan Hospital, Korea University, Dan Won-Gu, Ansan, GyoungGi-Do, Korea; Department of Internal Medicine, Ansan Hospital, Korea University, Dan Won-Gu, Ansan, GyoungGi-Do, Korea; Department of Internal Medicine, Ansan Hospital, Korea University, Dan Won-Gu, Ansan, GyoungGi-Do, Korea

**Keywords:** heparin, thrombocytopaenia, myocardial infarction

## Abstract

Heparin-induced thrombocytopaenia is a life-threatening complication, affecting the morbidity and mortality of the patient if not properly treated. We report a case of a 75-year-old female patient who experienced enoxaparininduced thrombocytopaenia during medical treatment of acute ST-segment elevation myocardial infarction due to thrombotic total occlusion in the large right coronary artery.

## Abstract

Thrombocytopaenia often occurs in critically ill patients. While there are many reasons for it, heparin-induced thrombocytopaenia (HIT) is one of the most fatal complications, characterised by the occurrence of thrombocytopaenia in conjunction with thrombotic manifestations after exposure to unfractionated heparin (UFH) or low-molecular-weight heparin (LMWH).^1^ The incidence of the HIT syndrome in patients exposed to heparin varies widely, depending on the preparation of the heparin and its concentration, varying from 0.2 to 5%.^2^

Clinical presentation of the HIT syndrome ranges from asymptomatic thrombocytopaenia to a variety of intravascular thromboses and embolisms after exposure to heparin. Thrombosis can affect both the arterial and venous system, however, venous thromboembolic complications are much more serious than arterial thrombotic events. Without alternate anticoagulation, the risk of thromboembolic complications can be seen in 30 to 75% of patients, and about 10 to 20% of patients suffer disseminate intravascular coagulation (DIC).^3^ The mortality rate associated with the HIT syndrome (HITS) is approximately 5 to 10%, usually secondary to thrombotic complications.^4^

The risk of HITS is higher in women and surgical patients compared with medical patients, and five- to 10-fold higher in patients receiving UHF compared to LMWH.^5^ Although rare, LMWH-induced thrombocytopaenia can occur and some cases have been reported in acute coronary syndrome. Here, we report a case of a patient who experienced enoxaparininduced thrombocytopaenia during medical treatment of acute myocardial infarction.

## Case report

A 75-year-old female visited the emergency department with chest pain of 12 hours’ duration. She had no other significant medical or family history except mild arthritis in both knees.

Her initial electrocardiogram showed a normal sinus rhythm with Q wave and ST-segment elevation in leads II, III and aVF. The echocardiogram demonstrated hypokinesia of the inferior wall of the left ventricle.

In the laboratory tests, the haemoglobin level was 12.7 g/dl, white blood cell count was 13.4 × 10^3^ cells/μl and the platelets were 302 × 10^3^ cells/μl. Initial coagulation studies showed a normal range. The initial level of CK-MB was 85.4 U/l and troponin-I was 20.2 ng/ml. Her clinical diagnosis was acute ST-segment elevation myocardial infarction of the inferior wall.

**Fig. 1.**
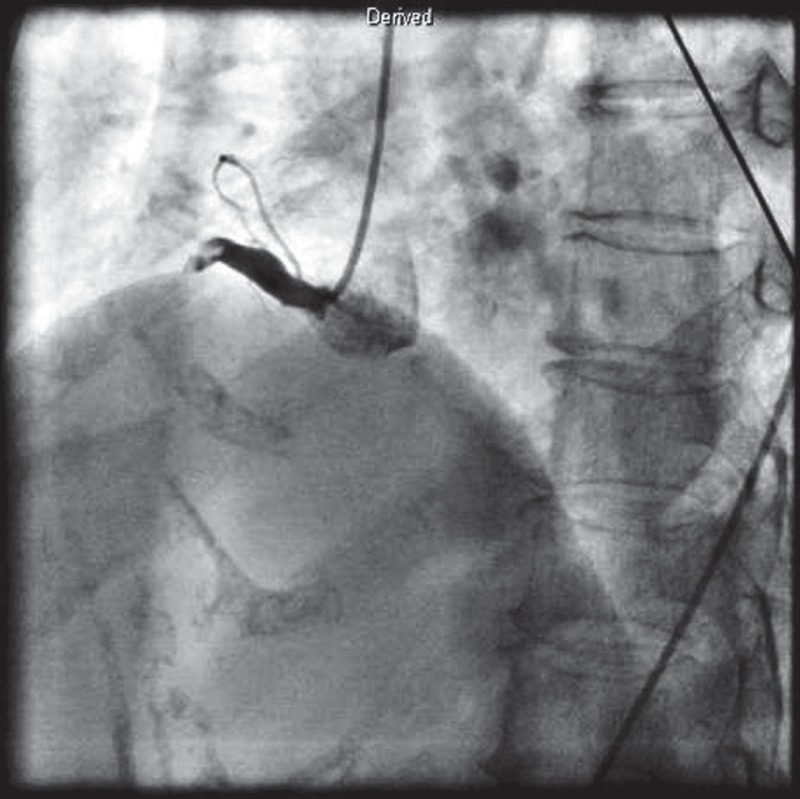
Emergent coronary angiogram (CAG) revealed total thrombotic occlusion of the proximal right coronary artery (RCA).

An emergent coronary angiogram (CAG) revealed total thrombotic occlusion of the proximal right coronary artery (RCA) (Fig. 1). The RCA was engaged with a 7-Fr guiding catheter (AL1, Cordis, Miami Lakes, Florida, USA) and predilatation was carried out with a Sprinter 3.0 × 20-mm balloon (Medtronic, Minneapolis, MN, USA) after a loadingdose injection of intracoronary abciximab.

After the procedure, the total thrombotic occlusion was still present, so we repeated thrombus aspiration with a thrombus extraction catheter (Thrombuster, Kaneka Medical Corp, Japan) and repeated the ballooning. The thrombotic occlusion did not improve and we decided on the second-stage procedure after one week of enoxaparin therapy in the intensive care unit. The patient was treated with aspirin, clopidogrel, statin and enoxaparin for one week.

**Fig. 2:**
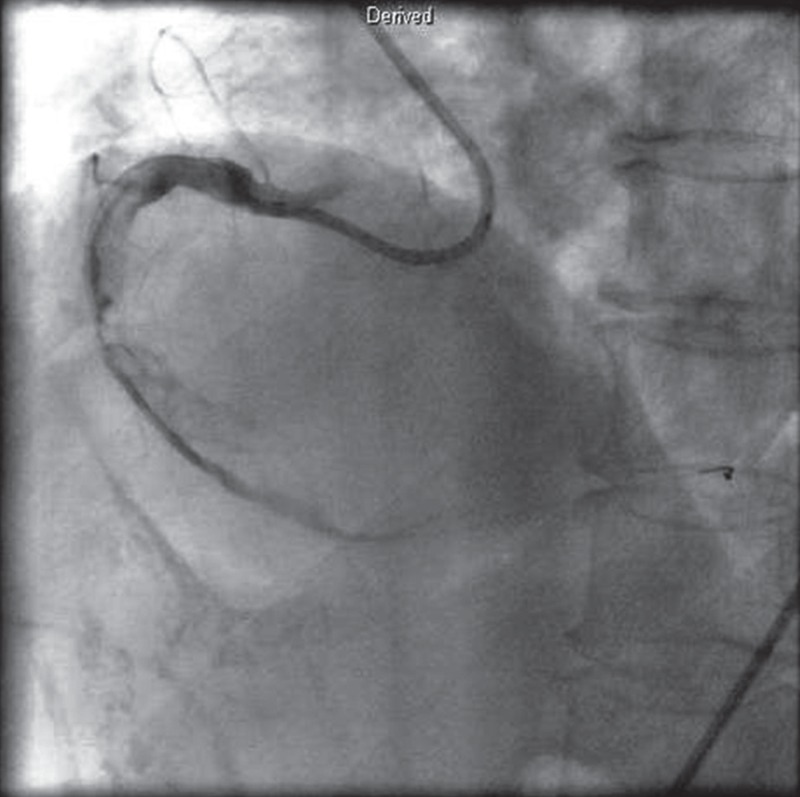
The follow-up CAG revealed thrombi still present in the large RCA, even after seven days of enoxaparin therapy.

**Fig. 3:**
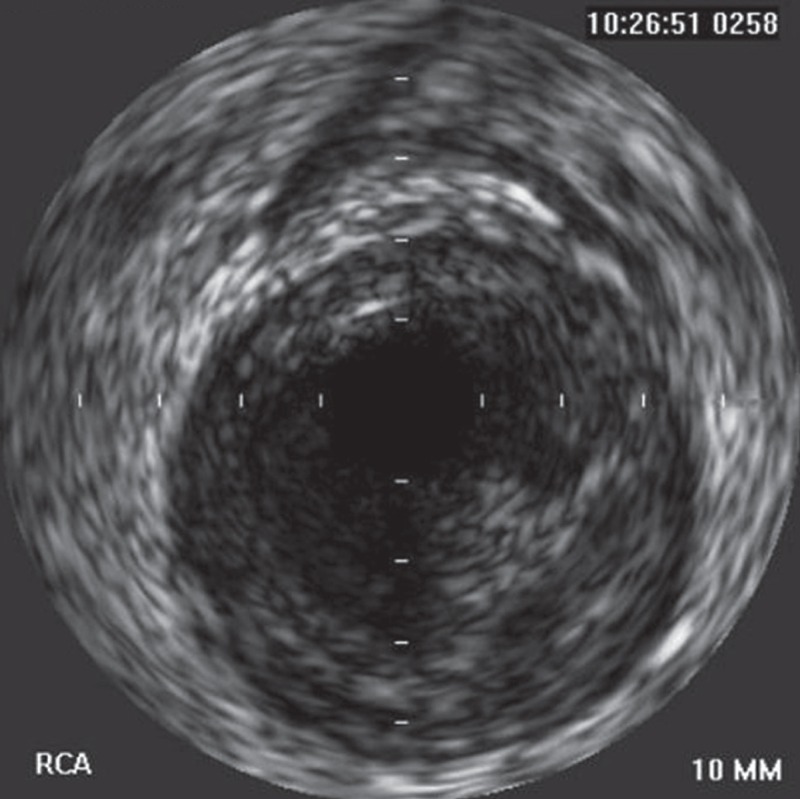
Intravascular ultrasound (IVUS) revealed thrombi still present in the large RCA. The reference diameter of the RCA was 6.2 mm.

A follow-up CAG (Fig. 2) and intravascular ultrasound (IVUS) were done after seven days of enoxaparin therapy, and it still revealed thrombi in the large RCA, despite the enoxaparin therapy. The reference diameter of the RCA was 6.2 mm (Fig. 3). The patient received repeated thrombus aspiration, but large thrombi still remained in the RCA. We decided to continue the enoxaparin therapy for several days instead of stenting, due to the large diameter of the RCA.

After 10 days of enoxaparin therapy, the patient complained of epistaxis and her platelet count was 11 000 cells/μl. We stopped the enoxaparin injection and checked the coagulation profile and heparin–platelet factor 4 antibody. The anti-factor Xa activity was not measured. The coagulation profiles were normal, but the heparin–platelet factor 4 antibody was strongly positive and she was a heparin-naïve patient. After discontinuation of enoxaparin, the platelet count recovered to 49 000 cells/μl on the first day and 117 × 10^3^ cells/μl on the second day. Fortunately, there were no adverse cardiac events.

**Fig. 4:**
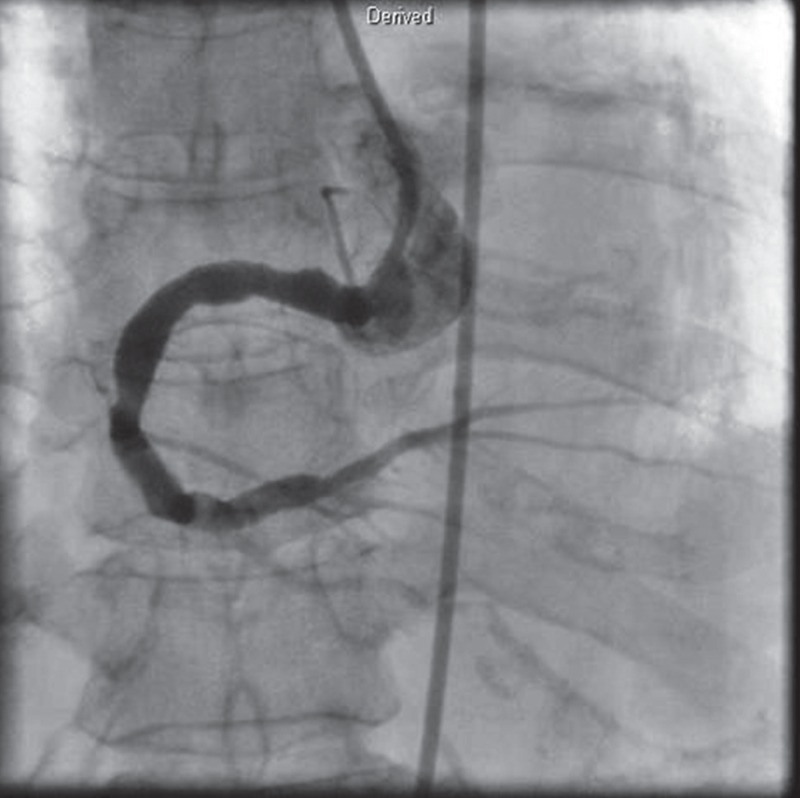
The follow-up CAG was performed on the 15th day after admission and revealed resolution of the thrombi in the RCA, with improved distal flow.

The second follow-up CAG and IVUS were performed on the 15th day after admission and revealed resolution of the thrombi in the RCA (Fig. 4). The patient was discharged and received medical therapy, including aspirin, clopidrogel and a statin instead of stent implantation. She had an uneventful recovery and there were no cardiac events during clinical follow-up of one year.

## Discussion

For over 80 years, heparin has been used clinically as an anticoagulant.^6^ Thrombocytopaenia as a result of heparin therapy was first described in the late 1960s.^7^ The HIT syndrome is characterised by thrombocytopaenia and thrombotic manifestations after exposure to heparin.^4^ Administration of heparin products is often in the setting of thrombosis or pro-thrombotic stimuli.

The initial steps of HITS involve patient exposure to heparin, followed by initial formation of the IgM antibody, and development of IgG antibodies over four to 14 days.^8^ IgG antibodies activate the platelets and release the contents of platelet granules. When platelet factor 4 (PF4) is released, it binds to heparin, resulting in a conformational change in PF4. The IgG antibodies and PF4–heparin become a ‘foreign antigen’, which can be immunogenic.^8^,^9^ The activated platelets secrete more PF4, feeding back to create more antigen and aggregate, which become procoagulant. Thrombin is then generated and platelet–fibrin thrombi are formed.^10^

When HITS appears to present with bleeding, this is usually the result of a thrombotic complication.^11^ For example, cerebral venous thrombosis causes increased venous congestion, and it may similarly manifest as intracranial haemorrhage. The thrombotic complications of HIT manifest as arterial or venous thromboses. Venous thrombotic events predominate over arterial events, and less common manifestations are necrotising skin lesions at the heparin injection sites.^12^

The severity of thrombocytopaenia is associated with higher risk of HIT-related thrombosis. In some HIT cases, there may be life-threatening complications, such as deep-vein thrombosis, pulmonary embolus, myocardial infarction, cerebral sinus thrombus, stroke, adrenal vein thrombosis, limb gangrene and acute limb ischaemia.^8^,^12^

The diagnosis of HITS includes a 50% fall in platelet count, beginning between five and 14 days after initial exposure to heparin of any dose or type, and detection of the HIT antibody against the PF4–heparin complex is necessary.^13^ PF4–heparin antibodies have been widely used for the diagnosis of HITS. The diagnostic criteria of HIT include thrombocytopaenia during heparin therapy, resolution of thrombocytopaenia after cessation of heparin, exclusion of other causes of thrombocytopaenia and confirmation of heparin-induced antibodies.^14^

There are two types in HITS.^15^ HIT type 1 is non-immunological and causes activation and aggregation of platelets, and eventually results in thrombocytopaenia. The degree of thrombocytopaenia does not fall below 100 000 cells/μl. It appears during the first hours of heparin administration and thrombosis is not observed. HIT type 2 is usually defined as a relative decrease in platelet counts to less than 50% of baseline or an absolute decrease to less than 100 000 cells/μl, typically five to 10 days after initiation of heparin therapy, a pattern indicative of the immunological aetiology of the condition.^16^

Compared to UFH, LMWH shows better outcomes, not only in thromboembolic events but also in complications such as HITS. Although antithrombotic therapy with LMWH is known to be safer than therapy with UFH, enoxaparin-induced thrombocytopaenia can occur.^5^ Even though enoxaparin-induced thrombocytopaenia occurred less often than HITS in one study, the clinical manifestations of both were similar.^17^

It is a general principle that for patients with suspected or confirmed HITS, all forms of heparin should be stopped and transfusion of platelet concentrate should not be considered unless thrombocytopaenia is life-threatening, or when the patient undergoes invasive procedures with high risk of bleeding, because the administered platelets would cause thromboembolic complications to develop or it would aggravate them.^13^,^14^,^18^ Anticoagulation with an alternative non-heparin anticoagulant should be commenced.

The direct thrombin inhibitors (DTIs) such as argatroban, bivalirudin and lepirudin are effective in the treatment of HIT-induced thromboembolism and as alternative anticoagulants for thrombosis prophylaxis in patients diagnosed with HIT.^15^,^16^,^18^ When DTIs are not available, factor Xa inhibitors such as fondaparinux should be administered. The binding of factor Xa inhibitors to antithrombin inhibits factor Xa, thus decreasing the rate of thrombin generation.^19^

In this case, we did not recognise any of these pathognomonical signs of enoxaparin-induced thrombocytopaenia, except a drop in platelet count and nasal bleeding after 10 days of anticoagulation therapy. The platelet count was normalised within days of discontinuation of enoxaparin.

Both the clinical situation of the patient and the medical treatment, including intracoronary abciximab, aspirin and clopidogrel could have been a cause of thrombocytopaenia, but the fact that the platelet count normalised after stopping enoxaparin, and the presence of anti-PF4–heparin antibodies suggested HITS. We diagnosed enoxaparin-induced thrombocytopaenia because of the clinical features, the patient’s heparin-naïve state and the laboratory finding of antibodies against PF4 and heparin complexes.

Another factor was the administration of aspirin and clopidogrel, which changed the activation of platelet aggregation in response to the stimulus of anti-PF4–heparin antibodies, although dual antiplatelet therapy with aspirin and clopidogrel neither treats HITS, nor aggravates HIT to form a thrombotic complication. This may explain why there was only nasal bleeding with the absence of any thrombotic complications in this patient, and it may have affected her prognosis.

We treated this patient with medication instead of stenting because of her large RCA diameter of more than 6 mm. The patient received dual antiplatelet agents, including 100 mg of aspirin and 75 mg of clopidogrel per day, and there were no other major adverse cardiac events during clinical follow up.

There are a few reports of enoxaparin-induced thrombocytopaenia in the literature but no reports however on enoxaparininduced thrombocytopaenia during medical treatment of acute myocardial infarction.

## Conclusion

We report our experience with enoxaparin-induced thrombocytopaenia during medical treatment of acute ST-segment elevation myocardial infarction. Although rare, LMWH such as enoxaparin may induce thrombocytopaenia, which could be a life-threatening complication. Physicians should always pay attention to complications such asb thrombocytopaenia, even when using LMWH.
